# Comprehensive Age-Stratified Impact of *NPM1* Mutation in Acute Myeloid Leukemia: A Real-World Experience

**DOI:** 10.3390/cancers17061020

**Published:** 2025-03-18

**Authors:** Vikram Dhillon, Abdul Moiz Khan, Jeff Justin M. Aguilar, Sushmitha Nanja Reddy, Mai M. Aly, Tariq Kewan, Waled Bahaj, Carmelo Gurnari, Valeria Visconte, David Carr, Julie Boerner, Jay Yang, Gregory Dyson, Jaroslaw Maciejewski, Suresh Kumar Balasubramanian

**Affiliations:** 1 Department of Oncology, Karmanos Cancer Institute, Wayne State University, Detroit, MI 48201, USA; 2 Department of Translational Hematology and Oncology Research, Taussig Cancer Institute, Cleveland Clinic Foundation, Cleveland, OH 44195, USA; 3 Clinical Hematology Unit, Department of Internal Medicine, Faculty of Medicine, Assiut University, Assiut 2074020, Egypt; 4 Department of Biomedicine and Prevention, Molecular Medicine and Applied Biotechnology, University of Rome Tor Vergata, 00133 Rome, Italy; 5 Department of Pathology, Wayne State University, Detroit, MI 48201, USA

**Keywords:** Acute myeloid leukemia, *NPM1*, *FLT3*-ITD, core-binding factor leukemia, ELN classification, AML prognosis

## Abstract

**Background:** While *NPM1*-mutated AML in the absence of *FLT3*-ITD generally carries a favorable prognosis, large registry studies suggest the positive prognostic benefit may not extend to patients > 65 years of age. We examined this preferential, age-dependent prognostic impact through a real-world analysis of 2811 adult AML patients. **Results:** The median overall survival (OS) was significantly better in *NPM1*^MT^ compared to *NPM1*^WT^ patients [20.86 vs. 17 mo., *p* = 0.003]. When stratified by age, *NPM1*^MT^ patients had higher OS than *NPM1*^WT^ patients in the 55–65-year age group (28.62 vs. 16.3 mo., *p* ≤ 0.0001). This OS benefit was heterogenous and prevailed most strikingly in the 55–60 (68.3 vs. 15.6 mo., *p* = 0.002), and up to the 60–65-year group (mOS not estimable vs. 20 mo., *p* = 0.007), but not beyond 65 y. Notably, the ≤65 cohort was more enriched with dominant *NPM1* (21% vs. 15%, *p* ≤ 0.001), while the >65 cohort was enriched with abnormal karyotype (20% in >65 years vs. 16% in ≤65 years, *p* = 0.001), and co-occurring *SRSF2* and *ASXL1* mutations (18.7% vs. 7.5%, *p* ≤ 0.0001 and 13.5% vs. 4.1%, *p* ≤ 0.0001 resp.). **Conclusions:** We demonstrate that in a real-world setting, the prognostic benefit of *NPM1* does not extend beyond age 65, underscoring the need for age-adapted risk stratification models. This granular approach could prevent the potential overestimation of prognosis in older patients with *NPM1*^MT^ AML and inform therapeutic decision making.

## Simple Summary

Acute myeloid leukemia (AML) is characterized by genetic mutations that influence disease course and treatment response. Mutations in the nucleophosmin (*NPM1*) gene are among the most common genetic abnormalities in AML and are generally associated with favorable outcomes. However, our comprehensive analysis of 2811 patients revealed that this prognostic benefit is strongly age-dependent. While *NPM1*-mutated AML patients younger than 65 years demonstrated significantly improved survival compared to their wild-type counterparts, this advantage disappeared in older patients. Our investigation identified several factors contributing to this discrepancy: older patients exhibited enrichment of adverse co-mutations, more frequently presented with abnormal chromosomal patterns, and received less intensive therapeutic interventions. Additionally, we found differences in the clonal dominance patterns of *NPM1* mutations between age groups. These findings underscore the necessity for age-adapted risk stratification in *NPM1*-mutated AML, which could prevent potential overestimation of prognosis in older patients and inform more appropriate therapeutic decision making, ultimately improving clinical outcomes.

## 1. Introduction

Somatic mutations in Nucleophosmin 1 or *NPM1* gene (*NPM1*^MT^) are earlier driver hits in leukemogenesis and one of the most commonly identified genetic abnormalities in de novo acute myeloid leukemia (AML; ~30% of cases) [1] The distinct molecular and clinical features of *NPM1*^MT^ AML led to its recognition as a separate entity in the 2017 World Health Organization (WHO) classification of myeloid neoplasms [2].

The prognostic significance of *NPM1*, particularly in conjunction with co-occurring mutations such as *FLT3-ITD* and myelodysplasia-related gene signature (AML-MR), has become increasingly central to risk stratification, as reflected in the 2022 European LeukemiaNet (ELN) guidelines [3,4,5]. Historically, *NPM1*^MT^ has been associated with favorable outcomes in AML patients [6,7]. Early observations from Falini et al. based on 591 AML patients showed that 291 *NPM1*^MT^ patients achieved a higher complete response (CR) rate and harbored less chemotherapy-refractory disease after induction therapy compared to *NPM1* wild-type *(NPM1*^WT^) [8]. Particularly, their work also highlighted that the favorable prognostic impact of *NPM1*^MT^ seemed limited to younger patients with median ages between 41.9 and 51.8. This age-related effect coincides with the observation that *NPM1*^MT^ occurs less frequently in older patients, who instead show enrichment for AML-MR. Subsequent analyses revealed stark survival differences between *NPM1*^MT^ patients above and below age 65 (median OS: 10.5 years vs. 1.7 years) [9].

Clinical trial-based registry studies have also suggested age as a strong prognostic indicator in *NPM1*^MT^ AML. More recently, it was discovered that the favorable prognostic impact of *NPM1*^MT^ was particularly pronounced in patients aged 55 to 65 years but diminished significantly in patients aged > 65 [10]. However, with age increasingly influencing treatment selection, the real-world implications of *NPM1*^MT^ age-dependent prognostic benefit remain poorly characterized outside of clinical trials [11]. Although it is conceived that chemotherapy without hematopoietic stem cell transplant is adequate for treating *NPM1*^MT^ AML, these non-uniform outcomes necessitate an exploratory analysis in a real-world cohort study. Accordingly, our work presents a comprehensive age-stratified analysis of a large cohort of *NPM1*^MT^ AML patients predominantly managed outside of conventional clinical trials to validate the differential prognosis and address the potential reasons underlying variable outcomes in this genetically defined subset of AML.

## 2. Methods

### 2.1. Cohort Selection

For this study, we used a large, well-annotated cohort of patients treated at Karmanos Cancer Institute (KCI) between 2006 and 2021 and expanded it using a publicly available dataset from cBioPortal and a meta-analytic registry (CCF) of AML patients from previously reported series [12]. More extensive details on the inclusion criteria for patients in the real-world meta-analytic registry are available elsewhere [12]. Patients with de novo AML were classified as primary (pAML), and patients with Myelodysplasia-Related Changes (MRC) or evolving from an antecedent myeloid neoplasm were classified as secondary AML (sAML). Due to a very small number of therapy-associated AML patients included in our analysis, this subgroup was also reclassified as secondary AML. Patients aged ≤65 and >65 years were classified as younger and older cohorts, respectively, for the purpose of this study. Patients were further subdivided into more granular age groups (<55, 55–60, 60–65, 65–75, and >75) in order to capture the age-related impact of *NPM1* mutation on overall survival. These cut-offs represent clinically meaningful inflection points in aging where disease biology, preexisting comorbidities, and treatment tolerability tend to shift overall survival and may otherwise be missed with broader age groupings. We analyzed the baseline clinical and molecular characteristics and performed survival analyses with respect to multiple genetic variables, focusing on the differences across the age groups. The clonal hierarchy of *NPM1*^MT^ in relation to other mutations was defined with a cut-off VAF difference of ≥5%. When the difference in VAF between the co-occurring mutations was less than 5%, the call was deemed codominant, as previously described [13].

Treatment was characterized as either intensive or non-intensive. Intensive therapies included high-dose cytarabine regimens with anthracyclines (such as the standard 7 + 3 regimen) or purine analogs (such as fludarabine, cladribine, or clofarabine). Non-intensive therapy primarily consisted of hypomethylating agents (azacitidine or decitabine) or low-dose cytarabine. Only a minority of patients received venetoclax, likely since it had not yet become the standard of care at the time of diagnosis for many of these patients. Additionally, The BEAT-AML Master trial dataset was used to validate the available data from KCI and CCF to study the impact of the treatment intensity [14].

### 2.2. Genetic Studies

For the genetic data generated from KCI, multiplex PCR of 568 amplicons was performed using the Illumina TruSight Myeloid Panel (Illumina, San Diego, CA, USA)to target frequently mutated regions of 49 myeloid genes. Next-generation sequencing was performed on Illumina’s MiSeqDx with 150 bp paired-end reads and a mean depth of coverage of 1000×. Sequencing data were analyzed with Illumina’s Local Run Manager software using the published human genome build UCSC hg19 as the reference sequence. Agilent’s Alissa Interpret 5.4 software (Agilent, Santa Clara, CA, USA) was used to evaluate sequence variations in individuals. All the reported sequence variants in patients with variant allele fractions less than 10% or 10–20% with <500× read depth were confirmed by Sanger sequencing. *FLT3*-ITD mutations and *CALR* exon 9 insertions or deletions were tested by multiplex fragment analysis. *CEBPA* sequencing was performed by Sanger sequencing for amplicons with less than 100x coverage by next-generation sequencing. Mutation nomenclature was based on the recommendations of the Human Genome Variation Society (HGVS). Variants were classified according to the Association for Molecular Pathology, American Society of Clinical Oncology, and College of American Pathologists standards and guidelines for interpreting and reporting sequence variants in cancer [15].

### 2.3. Statistical Analysis

Survival outcomes were assessed using the Kaplan–Meier methodology. Overall survival (OS) was defined as the time from initial diagnosis to death from any cause, with censoring at the date of last follow-up for surviving patients. Differences in survival curves were evaluated using the log-rank test. The reverse Kaplan–Meier method was employed to calculate the median follow-up time. For comparisons between groups, categorical variables were analyzed using the chi-squared test, while the Mann–Whitney test was used to compare medians of continuous variables. Statistical significance was set at *p* < 0.05 using two-tailed tests for all the analyses. All the statistical analyses were performed using the R statistical software (version 4.30), with Kaplan–Meier curves generated using GraphPad Prism 9.5.0.

## 3. Results

The patient clinical characteristics are listed in Table 1. We performed rigorous quality control on our entire cohort of 2811 patients and the remaining 2309 were further subdivided into *NPM1*^WT^ (*n* = 1820) and *NPM1*^MT^ (*n* = 489) (Supplementary Figure S1). The patient subgroup stratification is also detailed in the Supplemental Methods. Among the clinically relevant differences between the two groups, the *NPM1*^WT^ patients had a higher median age at diagnosis than *NPM1*^MT^ (*p =* 0.0006). The *NPM1*^WT^ group was more enriched with secondary AML (*p* = 0.0001), abnormal karyotype (*p* < 0.0001), and non-*FLT3*-ITD patients (*p* < 0.0001). Conversely, *NPM1*^MT^ had a hi gher proportion of primary AML (*p* = 0.0001), normal karyotype (*p* < 0.0001), and *FLT3*-ITD^POS^ (*p* < 0.0001) patients. *NPM1*^MT^ was also associated with a higher bone marrow blast percentage (*p* < 0.0001) and peripheral white blood cell count (*p* < 0.0001) at diagnosis.

We then explored survival outcomes based on various genetic variables and molecular profiles of our cohort.

### 3.1. Cohort Overview and OS Based on Co-Occurring Mutational Count

**Figure 1:**
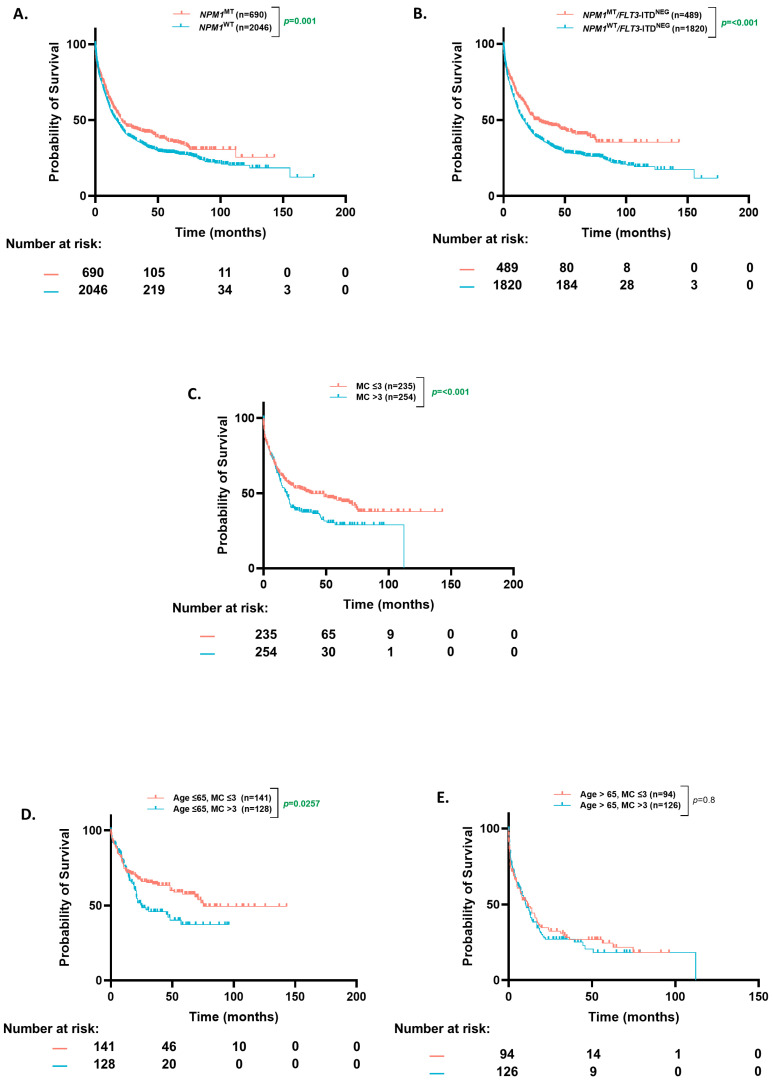
Overall survival based on *NPM1* status and mutational burden in patients ≤65 and >65 years. (**A**) Kaplan–Meier estimates of overall survival (OS) in all patients according to *NPM1* mutation status. (**B**) Kaplan–Meier estimates of OS in *NPM1*^MT^ patients based on *FLT3*-ITD^NEG^ status (**C**) Kaplan–Meier estimates of OS based on co-occurring mutation count in *NPM1*^MT^ patients. (**D**,**E**) Kaplan–Meier estimates of OS in *NPM1*^MT^ patients according to co-occurring mutation count [age ≤ 65 (**D**) and age > 65 (**E**)]. MC = mutation count.

In our entire cohort, the *NPM1*^MT^ patients had a significantly higher median overall survival (OS) than *NPM1*^WT^ (20.86 mo. vs. 17 mo., *p* = 0.003) (Figure 1A). The median follow-up time for our cohort was 45.6 months, and the 4-year OS reached 33.1% for the *NPM1*^WT^ patients (30.5–36.0%, 95% CI) and 43.5% for *NPM1*^MT^ (38.9–48.6%, 95% CI). When the *NPM1*^MT^ patients were stratified further based on ENL2022 favorable-risk criteria (*NPM1*^MT^ without *FLT3*-ITD), the *NPM1*^MT^ patients had a better OS compared to the wild-type population (29 mo. vs. 16 mo., *p* ≤ 0.0001) (Figure 1B).

A number of co-occurring mutations can predict clinical behavior in myeloid neoplasms [16]. Based on prior studies, we analyzed differences in OS of the *NPM1*^MT^ AML patients with respect to the number of co-occurring mutations (≤3 or >3). There was a higher proportion of patients with more than three mutations in the older age group (44% vs. 33%, *p* = 0.01). The patients with more than three than less than or equal to three co-occurring mutations had a worse OS (18 mo. vs. 38 mo. *p* < 0.0001) (Figure 1C). When stratified by an age cut-off of 65, the difference in OS based on mutation count was statistically significant in the younger (75 mo. vs. 25 mo., *p* = 0.026) (Figure 1D) but not the older patients (10.3 mo. vs. 11.3 mo., *p* = 0.8) (Figure 1E). However, most of the benefit in all comers was derived from the patients younger than 65.

### 3.2. Age-Stratified OS (Based on NPM1 Status)

**Figure 2:**
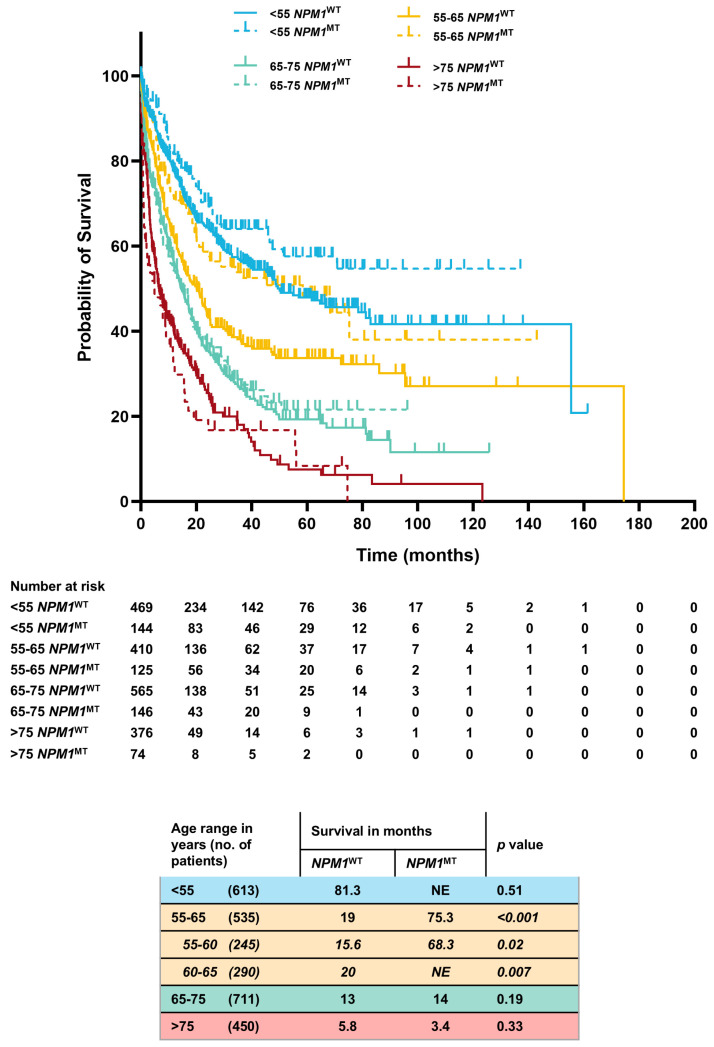
Comprehensive Kaplan–Meier estimates of OS stratified by age, *NPM1* mutation status, and *FLT3*-ITD^NEG^ genotype.

In order to understand the heterogeneity of the prognostic benefit provided by *NPM1*, we stratified the cohort (*n* = 2309) into clinically relevant age groups and observed significant differences. The *NPM1*^MT^ patients in the 55–65-year age group had a higher OS when compared to the *NPM1*^WT^ group (75.3 mo. vs. 19 mo., *p* ≤ 0.001). This benefit was preserved even if further sub-grouped into 55–60-year (68.3 mo. vs. 16 mo., *p* = 0.02) and 60–65-year (not estimable (NE) vs. 20 mo., *p* = 0.0007) age cohorts. In 65–75 and above-75-year groups, there was no difference in overall survival between the *NPM1*^MT^ versus *NPM1*^WT^ patients (65–75 years. age group, 13 vs. 14 mo., *p* = 0.19; >75 years. age group, 3.4 vs. 5.8 mo., *p* = 0.33). Patients younger than 55 years performed well regardless of the *NPM1*^MT^ status, and the *NPM1*^MT^ patients had a numerical survival advantage over *NPM1*^WT^ (NE vs. 81 mo., *p* = 0.51) (Figure 2).

### 3.3. Molecular Profile of NPM1^MT^ Patients

**Figure 3:**
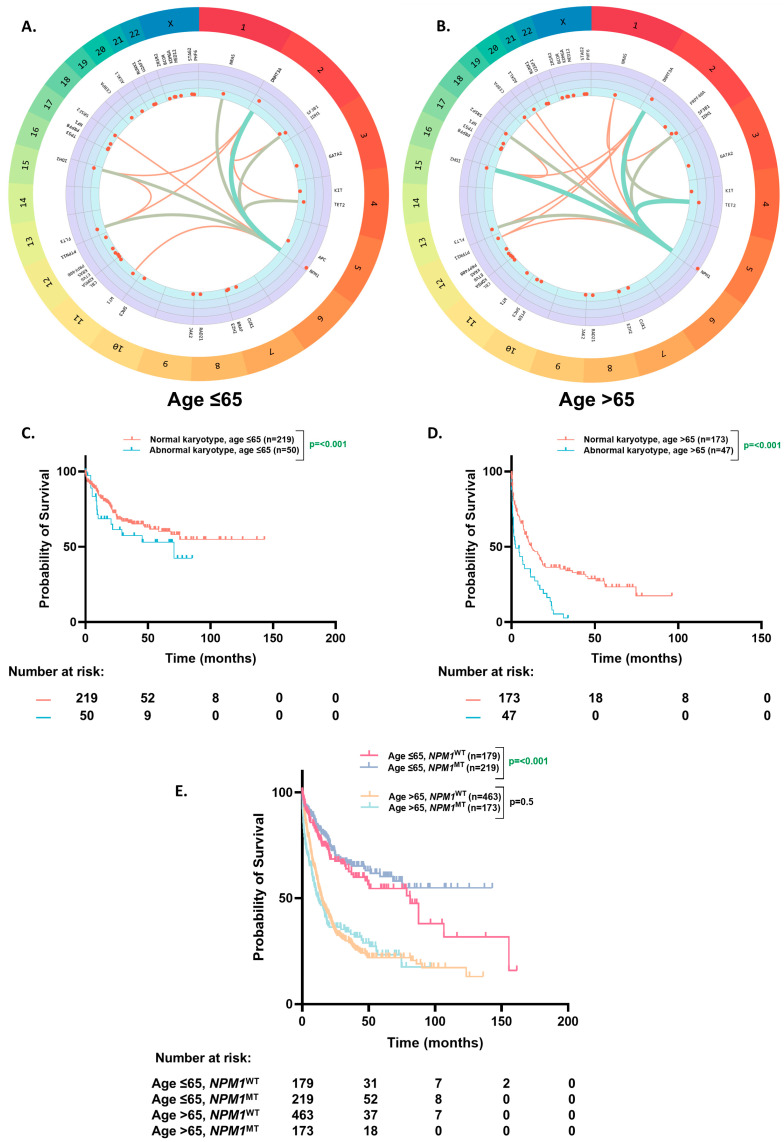
Co-occurring mutational profile and overall survival based on karyotype. (**A**,**B**). Circos plot illustrating the co-occurrence of somatic mutations in the *NPM1*^MT^ patients aged ≤ 65 (**A**) and aged > 65 (**B**). (**C**,**D**). Kaplan–Meier estimates of OS according to karyotype in the *NPM1*^MT^ patients aged ≤ 65 (**C**) and aged > 65 (**D**). (**E**). Kaplan–Meier estimates of OS based on the patients aged ≤ 65 and >65 and *NPM1* mutation status with a normal karyotype.

Among the co-occurring mutations with *NPM1*^MT^, the association of *DNMT3A* (46% vs. 39%, *p* = 0.27), *IDH2* (25% vs. 22%, *p* = 0.61), *FLT3*-ITD (31% vs. 19%, *p* = 0.16), *IDH1* (19% vs. 15%, *p* = 0.25), *PTPN11* (5% vs. 8%, *p* = 0.18), and *CEBPA* (5% vs. 6%, *p* = 0.63) were not different in the ≤65 vs. >65-year groups. *TET2* (13% vs. 27%, *p* = 0.0001)*, SRSF2* (5% vs. 15%, *p* = 0.0002), and *ASXL1* (1% vs. 7%, *p* = 0.0006) were more associated with *NPM1*^MT^ in the >65 years age group, whereas *NRAS* (13% vs. 7%, *p* = 0.03) and *WT1* (10% vs. 4%, *p* = 0.01) were more commonly seen in the ≤65 years cohort (Table 2, and Supplementary Figure S2). The strength of associations between these mutations was slightly different between the younger and older age groups (Figure 3A,B). We sought to investigate the impact of prognostically significant co-mutations with *NPM1* (*DNMT3A*, *FLT3-ITD,* and *WT1*) on overall survival in the younger (aged ≤ 65) and older (aged > 65) patients (Supplementary Figure S3). These three co-mutations have been previously recognized as defining a key archetype of *NPM1*^MT^ AMLs [16,17,18]. When *NPM1* was co-mutated with *DNMT3A*, OS was the most favorable among the triplet, and the younger patients had a significantly higher OS compared to the older ones (68 vs. 11 mo.; *p* ≤ 0.0001). Furthermore, in the younger patients, *WT1* co-mutation resulted in the next highest OS compared to the elderly (46 vs. 9 mo.; *p* ≤ 0.0001). In contrast, among the elderly patients, *FLT3*-ITD co-mutated with *NPM1* had the highest OS (Supplementary Figure S3; 21 mo. vs. 10 mo. for *DNMT3A* vs. 9 mo. for *WT1*; *p* ≤ 0.0001).

### 3.4. OS Based on Karyotype

A relatively higher proportion of the older patients had an abnormal karyotype than the younger patients (20% vs. 16%, *p* ≤ 0.001). The patients with an abnormal karyotype had worse OS than the patients with a normal karyotype in the younger (71 mo. vs. NE, *p* ≤ 0.0001) (Figure 3C) and older age group (2.4 mo. vs. 12 mo., *p* ≤ 0.0001) (Figure 3D).

On further analyses of survival in the patients with ELN2022 favorable risk (*NPM1*^MT^ without and cytogenetically normal AML (CN-AML)), the OS benefit of *NPM1* mutation was still restricted to the ≤65-year age group (NE vs. 38 mo., *p* = 0.001) and did not extend to the >65-year group (12 mo. vs. 14.6 mo., *p* = 0.5) (Figure 3E).

### 3.5. OS Based on Clonal Dominance

**Figure 4:**
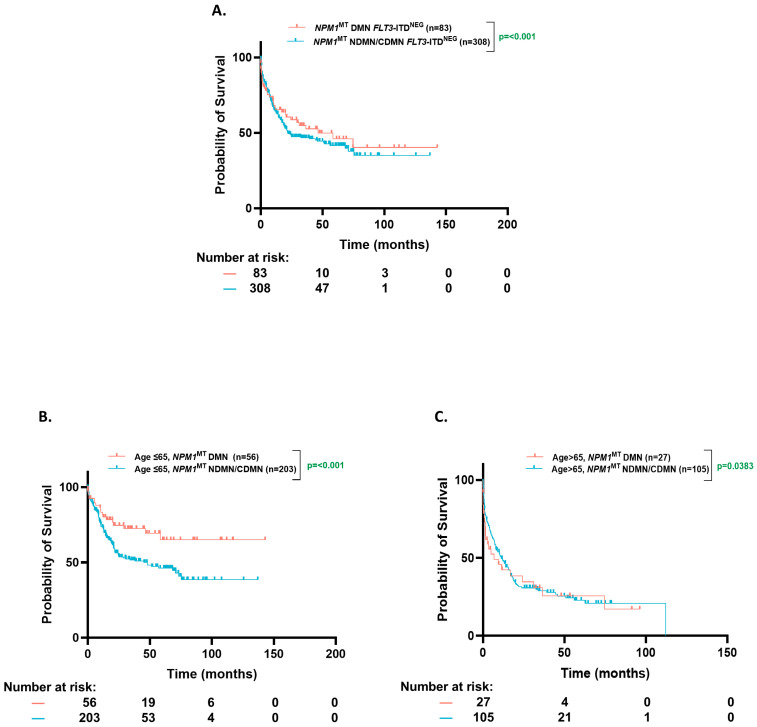
Overall survival based on clonal dominance. (**A**). Kaplan–Meier estimates of OS for all *NPM1*^MT^-dominant and *NPM1*^MT^-non-dominant/co-dominant patients according to Variant Allele Frequency (VAF). (**B**,**C**). Kaplan–Meier estimates of OS for *NPM1*^MT^-dominant and *NPM1*^MT^-non-dominant/co-dominant patients according to VAF [age ≤ 65 (**B**) and age > 65 (**C**)]. DMN= dominant; NDMN = non-dominant; CDMN = co-dominant.

The patients with a dominant *NPM1*^MT^ had a higher and statistically significant OS in all comers (74.6 mo. vs. 19.87 mo., *p* = 0.02), and *FLT3*-ITD^POS^ (NE vs. 16.8 mo., *p* ≤ 0.001) population, but not in *FLT3*-ITD^NEG^ (46.5 vs. 21.7 mo., *p* = 0.43), (Figure 4A).

The median VAF of *NPM1* was ~38% (Interquartile range (IQR), 30–45%) in both the younger and older cohorts. However, the ≤65-year group was more enriched with dominant *NPM1*^MT^ when compared to the >65-year group (28.8% vs. 18%, *p* = 0.01). The *NPM1*^MT^-dominant patients trended towards a better numerical OS in the ≤65-year group (NE vs. 75.2 mo. *p* = 0.21), whereas this trend was absent in the >65-year group (6.8 mo. vs. 10.3 mo., *p* = 0.6), although this comparison did not reach statistical significance (Figure 4B,C).

### 3.6. OS Based on Treatment Intensity

**Figure 5:**
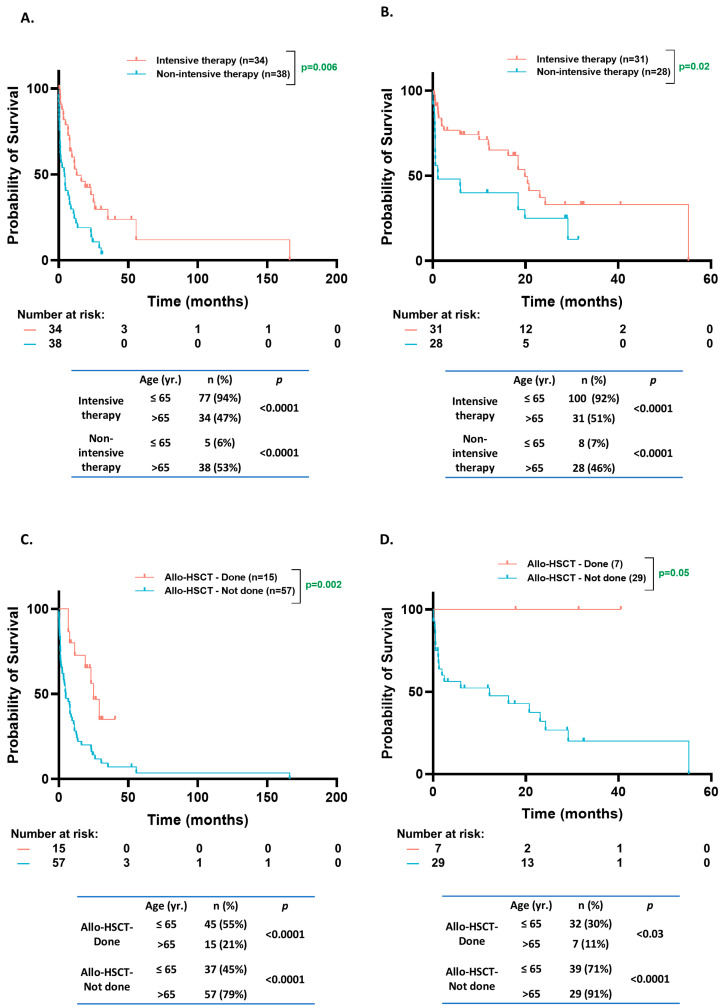
Overall survival based on treatment intensity and transplant status. (**A**,**B**). Kaplan–Meier estimates of OS according to treatment intensity of induction therapy for *NPM1*^MT^ patients from Karmanos Cancer Institute (KCI)/Cleveland Clinic Foundation (CCF) cohorts aged > 65 (**A**) and *NPM1*^MT^ patients from BEAT-AML and cBioPortal cohorts age > 65 (**B**). (**C**,**D**). Kaplan–Meier estimates of OS according to allogeneic stem cell transplant status for *NPM1*^MT^ patients from KCI/CCF age > 65 (**C**) and *NPM1*^MT^ patients from BEAT-AML cBioPortal cohorts age > 65 (**D**).

A higher proportion of patients received non-intensive induction therapy (53% vs. 6%, *p* < 0.0001) and a lower fraction received allo-HSCT (21% vs. 55%, *p* < 0.0001) in the older population (*n* = 72) compared to the younger age group (*n* = 82) in the CCF and KCI patient cohorts (Figure 5A,C). Only 4% of the younger patients had an Eastern Cooperative Oncology Group (ECOG) performance status score of ≥2 compared to 30% in the older population (*p* = 0.01). Comparable results were observed in the BEAT-AML dataset [non-intensive therapy in 46% vs. 7%, *p* < 0.0001; and allo-HSCT in 7% vs. 32%, *p* = 0.03; in older (*n* = 32) vs. younger (*n* = 55) patients, respectively] (Figure 5B,D). In the older group, the patients who received non-intensive therapy had a worse OS than those receiving intensive treatment (KCI/CCF cohort: 4.1 mo. vs. 13 mo., *p* = 0.0006; BEAT-AML cohort: 0.5 mo. vs. 23 mo., *p* = 0.03) (Figure 5A,B). Furthermore, the older patients who received allo-HSCT had a better prognosis than those who did not (KCI/CCF cohort: 24.9 mo. vs. 4.9 mo., *p* = 0.002; BEAT AML cohort: NE vs. 12.1 mo., *p* = 0.05) (Figure 5C,D).

## 4. Discussion

The survival benefit of *NPM1*^MT^ is age-dependent and lost in the older population. In a combined analysis of 743 patients with CN-AML treated in SWOG and MCR/NCRI trials, Ostronoff et al. demonstrated that *NPM1*^MT^, *FLT3-*ITD^NEG^, and CN-AML patients in the 55–65-year age group had a better two-year OS as compared to those without this genotype (65% vs. 40%, *p* < 0.001). On the contrary, patients in the >65-year age group had no significant improvement in 2-year OS (36% vs. 26%, *p* = 0.062), which was comparable to the findings in our largest comprehensive analysis of patients primarily treated outside of clinical trials [10,17].

*FLT3* is a strong predictor in determining the clinical course in AML, even in *NPM1-mutated* patients [17]. Besides *FLT3*, the difference in the molecular profile of the younger and older patients could partly explain the age-stratified impact on the prognosis. *SRSF2* and *ASXL1* mutations more frequently occur in the elderly, are associated with AML with MDS-related changes, and are recognized as adverse prognostic factors in the ELN 2022 risk classification [3,19]. On the other hand, the prognostic impact of *TET2*^MT^ is controversial [3,20,21]. *NRAS* and *WT1* mutations were more commonly seen in the ≤65 age group. Multiple studies show that *NRAS* mutations have no influence on prognosis [22,23]. Albeit *WT1* mutations seem to be associated with a poor prognostic impact, it is still unaccounted for in the current ELN classification [24,25,26]. More recent evidence suggests conflicting roles of secondary-type/MDS-related mutations on *NPM*1^MT^ AML where studies from Chan et al. and Zhao et al. suggest poor outcomes and Eckardt et al. suggest no impact on outcomes [18,27,28]. To ascertain the precise clinical relevance of secondary-type mutations in *NPM*1^MT^ AML and validate the impact on OS, larger registry studies are needed. Recent work by Othman et al. identified a prognostically significant archetypal co-mutation triplet in *NPM1*^MT^ AML characterized by *FLT3*-ITD, *DNMT3A*^MT^, and *WT1*^MT^ mutations [18]. While their study reported poorer outcomes (in terms of higher MRD and shorter OS) when *NPM1* co-occurred with any two mutations from this triplet, our analysis revealed that the negative impact was skewed towards the older compared to the younger patients. Notably, the younger *NPM1*^MT^ patients had a significantly improved OS when co-mutated with *DNMT3A* or *WT1*. This age-specific divergence in *WT1*’s prognostic significance is particularly noteworthy, as Othman’s cohort (derived from AML17 and AML19 trials) predominantly included patients under 60 years. Our findings provide external validation and important context for understanding the prognostic value of this co-mutational triplet in an older population.

The distinction between dominant and secondary mutant clones can have clinical implications for managing AML. Despite the inherent limitations in determining clonal hierarchy with VAF, it can still provide helpful information about the clonal architecture, prognosis, and response to treatment [29,30,31,32]. If clonal dominance alone sufficed a substantial prognostic impact, it would convey an unbiased effect on prognosis. However, we observed a numerically better OS with a dominant *NPM1*^MT^, and this potentially favorable impact was only limited to patients aged ≤ 65. In two studies of *NPM1*^MT^ AML patients, *NPM1* allelic burden in isolation did not correlate with prognosis, but clonal hierarchy was not described [33,34].

Intensive chemotherapy regimens are associated with higher rates of complete remission and more prolonged overall survival, particularly in younger AML patients or those with favorable risk factors [35,36]. However, other studies have found no significant difference in outcomes between intensive and less intensive treatment approaches, particularly in older patients or those with unfavorable risk factors [36]. Considering the varied treatment approaches for different age groups in *NPM1*^MT^ patients, we studied the impact of induction therapy and allogenic transplant at second remission on OS. The treatment differences between the age groups might have contributed to a lack of survival benefit of *NPM1*^MT^ in the elderly in our cohort. Ostronoff et al. described a lower CR rate and a higher one-year relapse rate but no difference in treatment-related mortality in the >65 vs. 55–65-year group, supporting a more intensive choice of therapy for the selected elderly patients [10]. In a UK NCRI study predominantly enrolling patients aged > 60 y, the CR rate in *NPM1*^MT^/*FLT3*-ITD^NEG^ was better than *NPM1*^WT^/*FLT3*-ITD^NEG^ patients with induction therapy, whether intensive (70% vs. 54%) or non-intensive (37% vs. 17%). However, the higher CR rate observed in the *NPM1*^MT^/*FLT3*-ITD^NEG^ patients with intensive and non-intensive induction therapy did not translate into a survival benefit [37]. A smaller study of patients older than 60 demonstrated similar findings, with the *NPM1*^MT^ patients receiving intensive induction therapy having a significantly higher CR rate than *NPM1*^WT^ (80.0% vs. 40.5% *p* = 0.03). At the same time, the OS was not statistically different [38]. One common limitation of these studies is the lack of information on subsequent allo-HSCT. For instance, Jentzsch et al. described a low relapse rate and favorable outcomes of older *NPM1*^MT^/*FLT3*-ITD^NEG^ AML patients consolidated with allogeneic stem cell transplant in CR1 [39].

While ECOG performance status does not provide a comprehensive assessment of a patient’s clinical fitness and comorbidities, a higher proportion of elderly patients in our data received non-intensive therapy, which cannot be explained by ECOG score alone [40]. Indeed, 53% received non-intensive treatment, but only 30% had an ECOG of ≥2. Further research is required to explore whether the choice of therapy in the older age group is guided by comorbidities or primarily determined by age-based stratification based on institutional practices and whether a careful selection of older patients for intensive chemotherapy may improve outcomes in this otherwise poorly performing population [41]. AML poses a significant treatment challenge, especially in the elderly. However, the guidelines recommend intensive antileukemic therapy in patients who can tolerate it [42,43]. Newer treatment options in AML, such as venetoclax in combination with hypomethylating agents (HMA), have shown improved outcomes in older *NPM1*^MT^ AML patients [44,45]. Menin interaction inhibitors such as revumenib and ziftomenib have been used in relapsed/refractory settings in *NPM1*^MT^- and *KMT2A*-rearranged AML, with complete or complete remission with a partial hematologic recovery rate (CR/CRh) of around 30% [46,47,48]. Whether these agents can bode more favorable outcomes in older patients with *NPM1*^MT^ AML remains to be seen. Menin interaction inhibitors in combination with other frontline agents currently being explored in clinical trials (NCT05735184, NCT04067336, NCT05848687, and NCT04065399) may shed more light on the outcomes [46,47,48].

We acknowledge the inherent selection bias related to any retrospective study. This notwithstanding, our study population broadly represents patients treated at different cancer centers and a snapshot of a “real-life” scenario. Comprehensive data on cytogenetic abnormalities for more granular classification into cytogenetic risk categories. Despite the attrition in our sample size due to missing molecular information, our analysis still provides a substantial age-stratified clinico-genomic report in *NPM1*^MT^ AML patients. Our analysis also emphasizes the need for better tools to assess patient “fitness”, providing a more meaningful objective translation of the subjective assessment to determine treatment decisions.

## 5. Conclusions

The favorable prognostic impact of *NPM1*^MT^ is lost in older people (>65 years) regardless of the cytogenetic background, which can be partially explained by differences in molecular profile and co-mutational patterns. Treatment selection significantly influences outcomes, and careful consideration of the clonal hierarchy of *NPM1* mutations may provide valuable insights when additional genomic alterations are present. Our findings suggest that age-adapted therapeutic approaches are crucial for the aging population. Improved risk stratification and patient selection for intensive chemotherapy versus allogeneic hematopoietic stem cell transplantation, optimized timing and dosing of venetoclax-based regimens, and the integration of emerging targeted therapies directed at *NPM1* or cooperating mutations may collectively enhance outcomes in elderly *NPM1*-mutated AML patients. Future prospective studies incorporating comprehensive age-stratified genomic profiling and minimal residual disease monitoring will be essential to develop personalized treatment algorithms for this biologically distinct subgroup of AML.

## Figures and Tables

**Table 1:** Patient characteristics.

Total Number of Patients (n = 2309)		*NPM1*^WT^ (n = 1820)	*NPM1*^MT^ (n = 489)	*p* Value
Median age at diagnosis—years. (range)		66.9 (9.9–100)	63.8 (20.2–91)	**0.0375**
Sex—n (%)	Female	960 (52.8%)	249 (50.9%)	0.49
	Male	860 (47.8%)	240 (49.1%)	
AML type—n (%)	pAML	1340 (73.6%)	461 (94.2%)	**0.0001**
	sAML	480 (26.4%)	28 (5.8%)	
Karyotype—n (%)	Abnormal	1178 (64.7%)	97 (19.8%)	**<0.0001**
	Normal	642 (35.1%)	392 (80.2%)	
ELN 2022 risk classification—n (%)	Favorable	169 (9.3%)	91 (18.5%)	
	Intermediate	724 (39.8%)	363 (74%)	**0.0001**
	Adverse	927 (50.9%)	35 (7.1%)	**0.0001**
Bone marrow blasts—% (range)		51.0 (2.5–99%)	73.0 (8.5–99%)	**<0.0001**
WBC count—×1000/mm^3^ (range)		5.65 (0–600)	25.6 (400–321)	**<0.0001**
Hemoglobin—g/dL (range)		9.3 (2.3–19)	9.2 (2.8–16.8)	0.69
Platelet count—×1000/mm^3^ (range)		55 (0–2366)	61.5 (7–493)	0.3109

pAML = primary AML; sAML = secondary AML.

**Table 2:** Distribution of mutations.

Mutation (n)	Age ≤ 65	Age > 65	*p* Value
*DNMT3A* (199)	107 (46%)	92 (39%)	0.27
*FLT3*-ITD (138)	81 (31%)	57 (19%)	0.16
*IDH2* (112)	58 (22%)	54 (25%)	0.61
*TET2* (91)	34 (13%)	57 (26%)	**0.0001**
*IDH1* (82)	49 (19%)	33 (15%)	0.25
*NRAS* (49)	33 (13%)	16 (7%)	**0.03**
*SRSF2* (44)	12 (5%)	32 (15%)	**0.0002**
*FLT3-TKD* (43)	24 (9%)	19 (9%)	1
*WT1* (33)	25 (10%)	8 (4%)	**0.01**
*PTPN11* (30)	12 (5%)	18 (8%)	0.18
*CEBPA* (24)	12 (5%)	12 (6%)	0.63
*ASXL1* (19)	3 (1%)	16 (7%)	**0.0006**
*KRAS* (19)	11 (4%)	8 (4%)	1

## Data Availability

The data that supports the findings of this study are available upon reasonable request.

## Supplementary Materials

The following supporting information can be downloaded at https://www.mdpi.com/article/10.3390/cancers17061020/s1, Figure S1: Patient selection; Figure S2: Co-existing mutations in *NPM1*^MT^ patients aged ≤ 65 and >65; Figure S3: *DNMT3A*, *WT1*, and *FLT3*-ITD co-mutations in *NPM1*^MT^ patients aged ≤ 65 and >65; Supplemental Methods.

## Abbreviations

The following abbreviations are used in this manuscript:

MDPI	Multidisciplinary Digital Publishing Institute
DOAJ	Directory of open access journals
TLA	Three letter acronym
LD	Linear dichroism
