# Supplementary material for: Comprehensive Age-Stratified Impact of NPM1 Mutation in Acute Myeloid Leukemia: A Real-World Experience
Source: Cancers (Basel). 2025 Mar 18;17(6):1020. doi: 10.3390/cancers17061020 (PMC11940789; doi:10.3390/cancers17061020)
Supplement: Supplementary file 1 [file cancers-17-01020-s001.zip › cancers-3476210-supplementary.pdf]

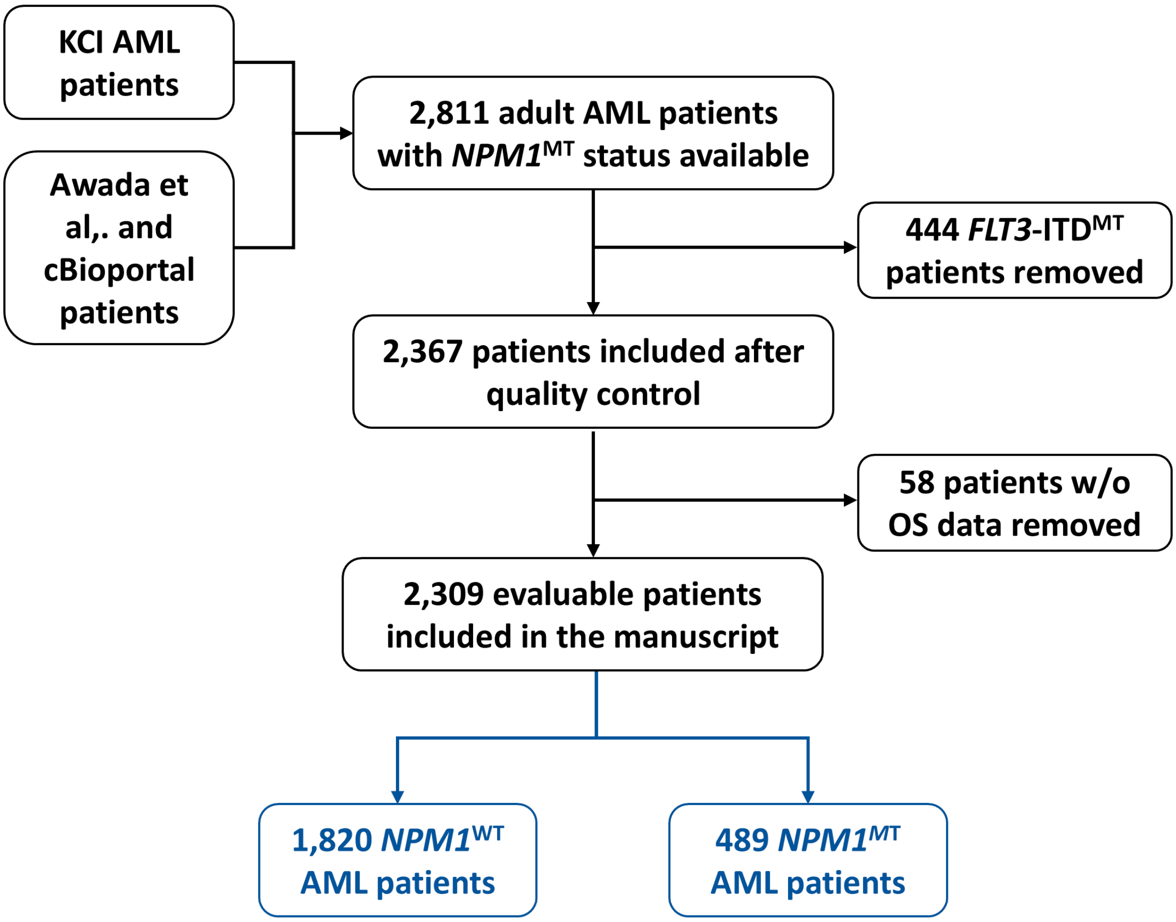

Figure S1. Patient selection

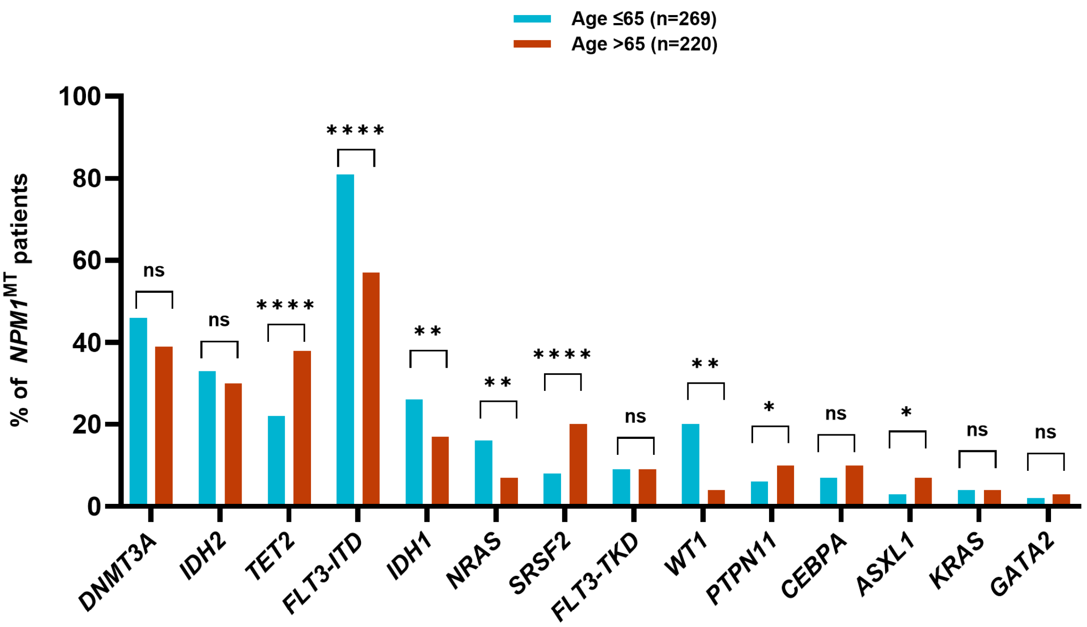

Figure S2. Co-existing mutations in *NPM1*^{MT} patients aged ≤65 and >65;.n.s., not significant; ****, $p < 0.0001$;**, $p<0.01$; *, $p < 0.05$.

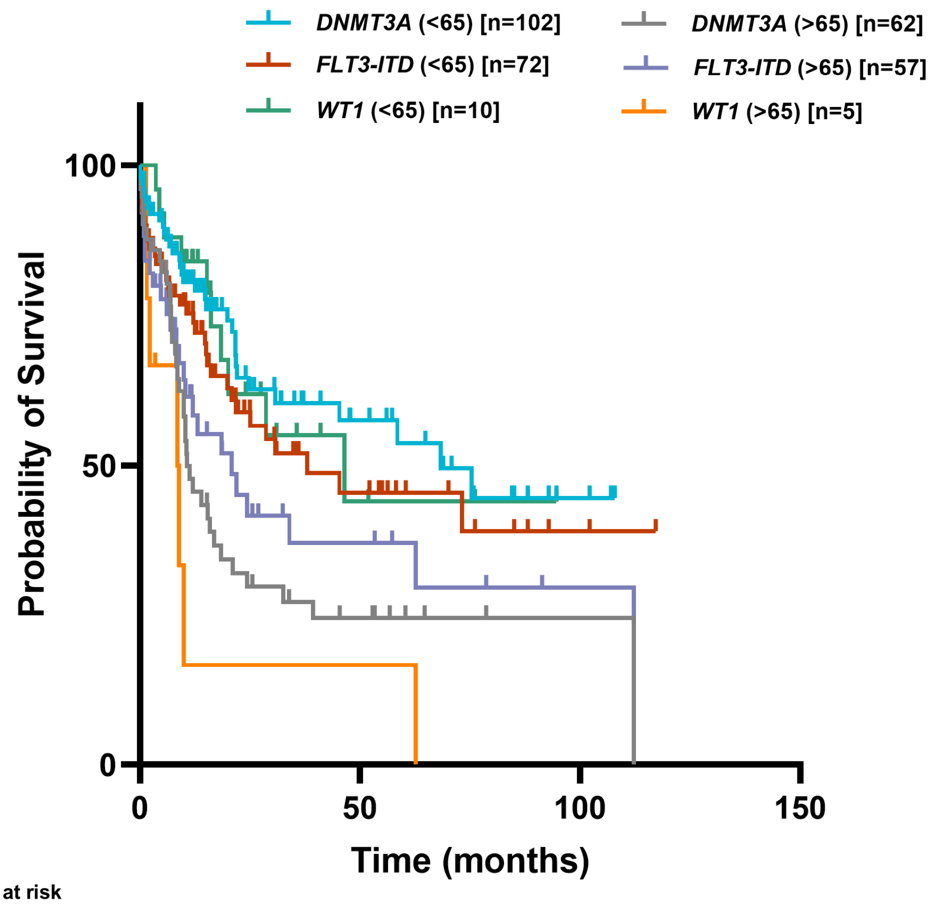

Number at risk				
DNMT3A (<65)	102	18	3	0
DNMT3A (>65)	62	8	1	0
FLT3-ITD (<65)	72	14	2	0
FLT3-ITD (>65)	57	8	1	0
WT1 (<65)	10	4	0	0
WT1 (>65)	5	1	0	0

Figure S3. *DNMT3A*, *WT1*, and *FLT3*-ITD co-mutations in *NPM1*^{MT} patients aged ≤65 and >65.

Supplemental Methods:

Our cohort consisted of 2,811 patients with evaluable *NPM1* mutational status. To account for the known adverse prognostic influence of *FLT3*-ITD mutations in *NPM1*-mutated patients across all age groups, we excluded 444 *FLT3*-ITD^{MT} patients. Among the remaining 2,367 patients, 58 additional patients were removed due to missing overall survival (OS) data, resulting in a final cohort of 2,309 patients who were stratified based on

NPM1 mutational status. This cohort selection process is visually represented in **Supplemental Figure S1**.

Real-world data from clinical settings frequently exhibits substantial missingness, which presents analytical challenges distinct from controlled clinical trials. In our cohort, 2.45% (58/2,367) of eligible patients lacked overall survival data after initial exclusions. Additionally, molecular characterization was incomplete for numerous patients, with variable rates of missingness across different genetic markers. This pattern of incomplete data is consistent with previously reported real-world AML studies, where missingness rates of 5-20% for key clinical variables and up to 30-40% for comprehensive molecular profiling are commonly observed, reflecting the constraints of retrospective data collection.

In **Figure 1A**, we present the OS analysis for the entire cohort in relation to *NPM1* mutational status, with OS data unavailable for 75 patients in total. All subsequent analyses and figures in this manuscript are based on the quality-controlled cohort of 2,309 patients. Within the final dataset, 489 patients were *NPM1*^{MT} including 269 patients 65 years and younger, as well as 220 patients over the age of 65. Among the *NPM1*^{WT} patients, 879 were 65 and younger, while 941 were older than 65 years. Finally, 28% of the *NPM1*^{MT} patients were missing a VAF for the *NPM1* mutation, and we are unable to determine the dominant vs co-dominant or non-dominant status.
